# Bisphenol A, phthalate metabolites and glucose homeostasis in healthy normal-weight children

**DOI:** 10.1530/EC-17-0344

**Published:** 2017-12-13

**Authors:** Amalie Carlsson, Kaspar Sørensen, Anna-Maria Andersson, Hanne Frederiksen, Anders Juul

**Affiliations:** 1 Department of Growth and ReproductionRigshospitalet, University of Copenhagen, Copenhagen, Denmark; 2 International Research and Research Training Center in Endocrine Disruption of Male Reproduction and Child Health (EDMaRC)Copenhagen, Denmark; 3 The Child and Youth ClinicRigshospitalet, University of Copenhagen, Copenhagen, Denmark

**Keywords:** endocrine-disrupting chemicals, children, OGTT, obesity, diabetes

## Abstract

**Introduction:**

Bisphenol A and several of the most commonly used phthalates have been associated with adverse metabolic health effects such as obesity and diabetes. Therefore, we analyzed these man-made chemicals in first morning urine samples from 107 healthy normal-weight Danish children and adolescents.

**Method:**

This was a cross-sectional study. Participants were recruited as part of the Copenhagen Puberty Study. The subjects were evaluated by an oral glucose tolerance test (OGTT), a dual-energy X-ray absorptiometry (DXA) scan, direct oxygen uptake measurement during cycle ergometry and fasting blood samples. First morning urine was collected and phthalate metabolites and BPA were measured by liquid chromatography-tandem mass spectrometry (LC–MS/MS) with prior enzymatic deconjugation. Individual chemical concentrations were divided into tertiles and analyzed in relation to biological outcome.

**Results:**

Children in the lowest tertile of urinary BPA had significantly higher peak insulin levels during OGTT (*P* = 0.01), lower insulin sensitivity index (*P* < 0.01), higher leptin (*P* = 0.03), triglyceride (*P* < 0.01) and total cholesterol levels (*P* = 0.04), lower aerobic fitness (*P* = 0.02) and a tendency toward higher fat mass index (*P* = 0.1) compared with children in the highest tertile for uBPA. No significant differences in anthropometrics, body composition or glucose metabolism were associated with any of the phthalate metabolites measured.

**Conclusion:**

This pilot study on healthy normal-weight children suggests an inverse association between BPA and insulin resistance. Our findings contrast other cross-sectional studies showing a positive association for BPA, which may be due to confounding or reverse causation because diet is an important source of both BPA exposure and obesity.

## Introduction

Concerns have been raised that man-made chemicals such as phthalates and bisphenol A (BPA) may have endocrine properties in humans. These potential endocrine disruptive chemicals (EDCs) are widely used in modern societies, and the majority of the human population is exposed to several of these environmental chemicals daily (1).

BPA is an estrogenic high-volume chemical used as the base compound in the manufacturing of polycarbonated plastics and epoxy resins, which are used for a variety of plastic products and barrier coatings for the inner surfaces of food and beverage cans (2). Humans are primarily exposed to BPA through ingestion (3). Urinary BPA excretion has been positively associated with higher prevalence of diabetes (4) and obesity (5) in adults and obesity (6) in children.

Phthalates are used as softeners in PVC plastics. As phthalates are continuously emitted from PVC, humans can be exposed through ingestion, inhalation or by dermal contact (7). Serious adverse health effects have been associated with high phthalate exposure such as alteration in reproductive development and increased risks of insulin resistance and obesity in human adults (8, 9, 10, 11, 12). Importantly, children may be at increased risk of exposure as phthalates are used in the manufacturing of many industrial products such as toys, building materials and food packaging (7). Despite increased regulations regarding the use of phthalates in children’s toys, detectable measures of phthalate metabolites are still being found in 97% of the childhood population (1). The exposure to phthalates has been associated with increased risk of obesity in childhood, but data are equivocal (6, 13, 14). As for BPA, human data on effects of phthalates on insulin sensitivity are still lacking.

In the present study, urinary excretion of BPA and metabolites of the most commonly used phthalates in 107 healthy normal-weight Danish children were associated to fasting lipids, leptin and adiponectin levels as well as to glucose and insulin levels during a two-h oral glucose tolerance test (OGTT).

## Methods

### Participants

Subjects were recruited as part of the Copenhagen Puberty Study (15, 16) from primary schools in the Copenhagen community. A total of 107 healthy Caucasian children (58 girls) aged 8.5–16.1 years volunteered. Characteristics of the children are specified in Table 1. All participants had total body fat and lean mass evaluated with a whole-body dual-energy X-ray absorptiometry (DXA) scan using a CDR 1000/W densitometer (Hologic, Bedford, MA, USA) with software, version 6.2. Fat mass index was calculated as total fat mass divided by height squared. Aerobic fitness was evaluated by assessing maximal oxygen uptake (V_O2_max) during a cycle ergometry test using an electronically braked cycle ergometer (Ergomedic 839: Monark, Varberg, Sweden). V_O2_max was measured directly using an online pulmonary gas analyzer system (Quark CPET; Cosmed, Rome, Italy). Data on OGTT, body composition as well as other aspects have previously been published (17, 18). The study was approved by the local ethics committee (reference no. KF 01 282214 and KF 11 2006-2033). Consent has been obtained from each patient or subject after full explanation of the purpose and nature of all procedures used.

**Table 1:** Basic characteristic of the 107 subjects included in the study.

	Boys (*n* *=* 49)	Girls (*n* *=* 58)
BMI (kg/m^2^)	18.07 (15.0–26.1)	18 (14.4–28.3)
Fat mass index (kg/m^2^)	3.2 (1.4–6.8)	3.6 (2.0–9.0)
Fasting glucose (mmol/L)	4.9 (3.9–5.9)	4.8 (3.2–5.4)
2-h glucose (mmol/L)	5. 1 (3–7.3)	5.1 (3.3–7.3)
Fasting insulin (pmol/L)	42 (10–102)	50 (11–168)
Peak insulin (mmol/L)	352 (112–1004)	407 (150–1329)
Triglycerides (mmol/L)	0.6 (0.4–1.7)	0.8 (0.4–2.0)
HDL (mmol/L)	1.5 (1.1–2.1)	1.5 (0.7–2.2)
LDL (mmol/L)	2.1 (0.5–3.2)	2.3 (0.8–3.8)
Total cholesterol (mmol/L)	3.6 (2.5–4.6)	3.7 (2.6–5.6)
Leptin (ng/mL)	3987 (884–37065)	6549 (1772–49495)
Adiponectin (μg/mL)	24532.5 (12100–57085)	27320 (9375–60190)
V_O2_max (mL/kg/min)	46.7 (30.3–63.2)	40.3 (25.1–49.3)

Data are expressed as medians and range.

### Blood sampling

Venous fasting blood samples were drawn after 12 h of fasting from the ante-cubital vein into standard vacuum tubes and centrifuged (3000 ***g*** at 10 min) within 30 min. Plasma was immediately stored at −20°C until analysis. A standard two-h OGTT with an oral glucose load of 1.75 g of glucose per kilogram bodyweight (maximum 75 g glucose) was performed for all subjects. Blood samples were drawn with 30-min intervals for determination of glucose and insulin levels. Insulin sensitivity index a.m. Matsuda was calculated. In addition, fasting leptin, adiponectin and lipid profiles (triglyceride, total cholesterol and high- and low-density lipoproteins) were determined as previously described (17).

### Urine sample collection and chemical analysis

Each of the participants collected two consecutive first morning urine samples and one 24-h urine sample. All samples were collected in November 2007 and have previously been analyzed for the content of metabolites of the most common phthalate diesters; diethyl phthalate (DEP), di-n-butyl phthalate (DnBP), di-iso-butyl phthalate (DiBP), butylbenzyl phthalate BBzP, di-(2-ethyl-hexyl) phthalate (DEHP) and di-iso-nonyl phthalate (DiNP) and BPA as one out of nine environmental phenols measured by liquid chromatography-tandem mass spectrometry (LC–MS/MS) with prior enzymatic deconjugation (19, 20).

For the present study, first morning urine collected from the first of the two consecutive mornings was used.

### Data analysis

The volume of urine was determined by converting the weight in grams of the collected urine into milliliters, as the urinary density is close to 1 g/mL (varies between 1.003 and 1.035 g/mL). Urinary concentrations of phthalate metabolites (ng/mL) were measured and converted to absolute amount excreted (µg per first morning urine sample) by multiplying the urinary concentration in each sample with the urine volume (mL).

The metabolite concentration (ng/mL) of DiBP and DnBP were summed (∑MBP_(i+n)_) and in order to combine the phthalate metabolites with different molecular weight, the amount of each metabolite was converted into molar concentration, summed in groups and back-converted into ng/mL by multiplying with the molecular weight of DEHP for the sums of four DEHP metabolites (∑DEHPm) and the molecular weight of DiNP for the sums of four DiNP metabolites (∑DiNPm). The molar sum of all measured phthalate metabolites (∑all phth.m) were not back-converted but expressed in µM.

### Statistics

Urinary BPA, monoethyl phthalate (MEP), ∑MBP_(i+n)_, monobenzyl phthalate (MBzP), ∑DEHPm, ∑DiNPm and ∑all phth.m were log-transformed to assure a Gaussian distribution. Correlations between the summed phthalates and BPA levels were evaluated by Pearson Correlations.

The compounds were divided into tertiles allowing children with urinary excretion levels below the limit of detection (LOD) to be included. Differences in metabolic parameters between tertiles were evaluated by independent Student *t*-tests. Peak insulin levels, insulin sensitivity index, triglyceride levels, fat mass index and leptin levels were all log-transformed before analyses. Univariate ANOVAs (general linear models) were used for adjusted analyses with sex and pubertal stage (3 groups: pre-pubertal, breast/genital stage 2–3 and breast/genital stage 4–5) included as fixed variable and age as continuous variable. No significant interactions were found between sex and puberty in any of the reported models except for insulin sensitivity for which the results of the combined as well as the sex-specific analyses is shown. Potential confounders divided into BPA tertiles are shown in Table 2. All statistical analyses were done using the statistical software IBM SPSS, version 23.0.

**Table 2:** Potential confounders divided into the BPA tertiles.

	Low (*n* *=* 35)	Medium (*n* *=* 35)	High (*n* *=* 37)
Boys/Girls (*n*)	16/19	16/19	17/20
Age (years)	12.3 (8.7–15.4)	12.4 (8.5–15.4)	12.3 (8.7–16.1)
Puberty (stage*)	8; 13; 14	7; 12; 16	12; 11; 14
BPA (ng/mL)	1.03 (<LOD–1.74)	2.55 (1.38–4.11)	6.83 (3.82–17.32)

*Breast/genital stage l; breast/genital stage 2–3; breast/genital stage 4–5.

## Results

In total, 107 children were included in the analyses. One-hundred and four children (55 girls) completed a full 2-h glucose tolerance test, and aerobic fitness levels were available in 91 (48 girls). Basic characteristics of all children are shown in Table 1. None of the children had an abnormal glucose tolerance test. Eight children (5 girls) had urinary BPA below LOD (<0.12 ng/mL). Median and range of values of measured phthalate metabolites and BPA are shown in Table 3. All phthalate metabolites were positively correlated (*r* > 0.35; *P* < 0.01). BPA did not correlate with neither of the measured phthalate levels (*r* < 0.08; *P* > 0.43).

**Table 3:** Levels of measured phthalate metabolites and BPA in the 107 children and adolescents included in the study.

	*n*	Median	Range
MEP (ng/mL)	107	46.822542	3.6893–658.24
MBP (ng/mL)	107	190.09819	48.1209–713.1561
MBzP (ng/mL)	107	32.930963	3.0639–451.7129
∑DEHP (ng/mL)	107	167.5	50.5–771.5
∑DiNP (ng/mL)	107	31.1	6.6–673.3
∑corr Phth.m (µM) (nmol/mL)	107	2.054935	0.583–5.5409
∑alll Phth.m (µM) (nmol/mL)	107	1.627242	0.4334–5.0396
BPA (ng/mL)	107	2.76	<LOD–17.32
BP-3 (ng/mL)	107	1.484424	<LOD–136.4388
∑DCP (ng/mL)	107	0.93621	<LOD–23.3397

BPA, bisphenol A; MEP, monoethyl phthalate; MBP, monobutyl phthalate isomers; MBzP, monobenzyl phthalate; ∑DEHPm, sum of DEHP metabolites; ∑DiNPm, sum of DiNP metabolites; ∑all phth.m, sum of all phthalate metabolites (MEP, MiBP, MnBP, MBzP and metabolites of DEHP and DiNP).

**Figure 1:**
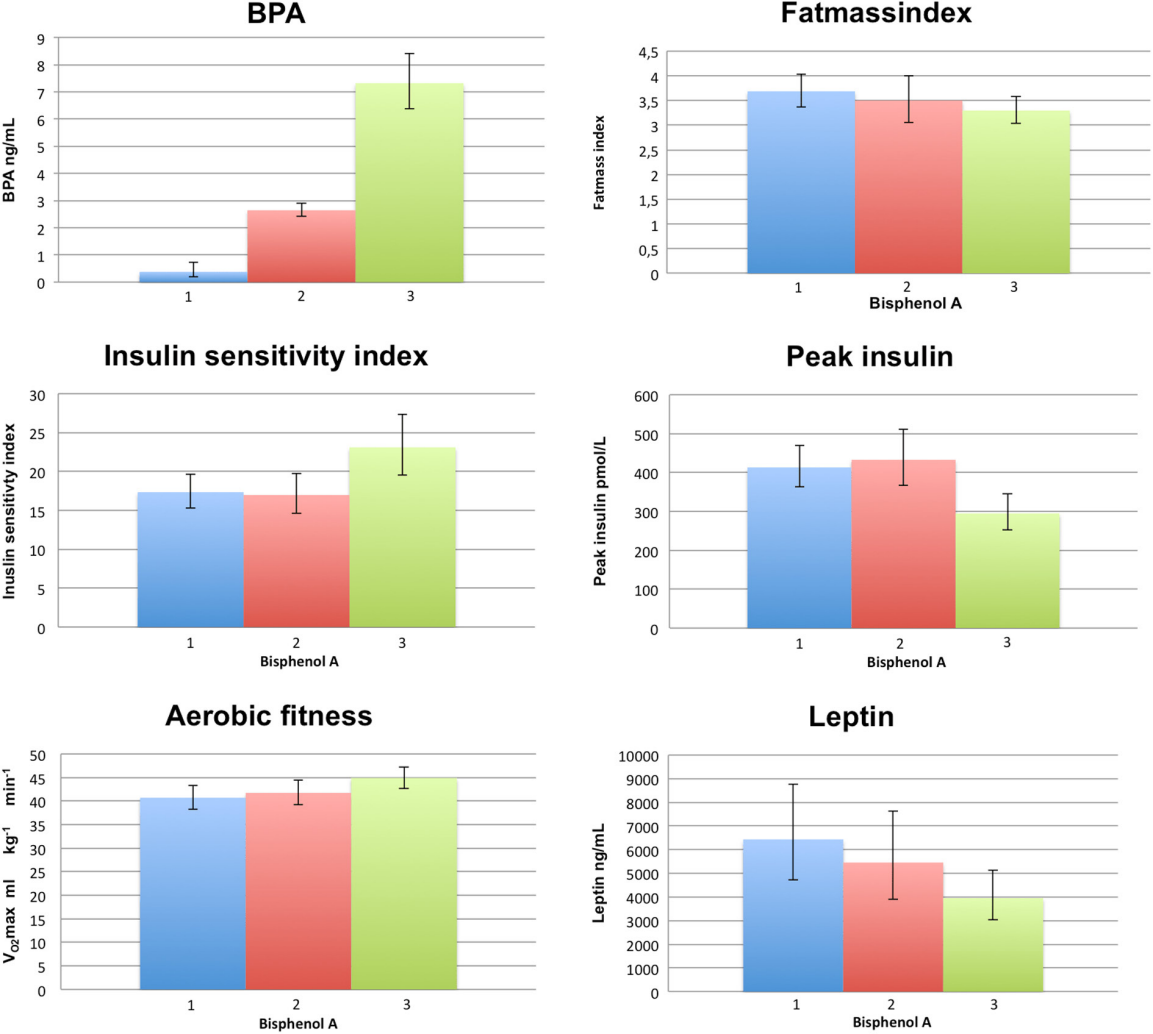
Bisphenol A (BPA) tertiles and the distribution of selected metabolic parameters within BPA tertiles.

The lowest tertile of BPA had significantly higher peak insulin levels during OGTT (*P* = 0.01), lower insulin sensitivity index (*P* < 0.01), higher leptin (*P* = 0.03), triglyceride (*P* < 0.01) and total cholesterol levels (*P* = 0.04), lower aerobic fitness (*P* = 0.02) and a tendency toward higher fat mass index (*P* = 0.1) compared with the highest tertile for BPA (Fig. 1). No differences were found for adiponectin or lipoprotein levels between the BPA tertiles (all *P* > 0.2). Sex, age and pubertal development were equally distributed among tertiles (*P* > 0.4). Adjusted for sex, age and pubertal development, the highest BPA tertile was still significantly associated with lower peak insulin levels (28.7% (5.1–57.5), *P* = 0.02), higher insulin sensitivity index (22.1% (2.2–45.8), *P* = 0.03) and lower triglyceride levels (25.4% (5.5–48.9), *P* = 0.03) compared with the lowest BPA tertile (ANOVA). Due to the significant interaction between sex and pubertal development for insulin sensitivity index, sex-specific analyses were done. Although none of these analyses were significant, the parameter estimates were similar to the combined analysis (boys: 19.8% (−6.0 to 52.7), *P* = 0.14; girls: (26.5% (−2.6 to 64.4), *P* = 0.08)). Additional adjustment for fat mass index did not change any of the results in the adjusted analyses (not shown). In adjusted analyses, no associations were found between BPA tertile and fat mass index or fitness levels, respectively (*P* > 0.16).

No significant differences in anthropometrics, body composition or glucose metabolism were found between tertiles of any of the phthalates measured (results not shown).

## Discussion

In this clinical study of healthy normal-weight children, urinary BPA and phthalate metabolites were present in detectable levels in the majority of urine samples, but no association was found between current exposure to these chemicals and adverse effect on body composition or glucose metabolism. On the contrary, we found that the group of children with the highest urinary excretion of BPA had significantly lower glucose-stimulated peak insulin levels, better insulin sensitivity and lower triglyceride levels compared with the lowest exposed children. These results were independent of adiposity.

It is well documented that the general population and children in particular are exposed to manufactured chemicals such as phthalates and BPA (1, 21). The more important question is whether these exposures form a relevant health risk. The hypothesis that exposure to these chemicals during childhood predispose to obesity and metabolic disease is far from verified (5, 6, 22).

To shed more light on this issue, we aimed to evaluate the dynamics in glucose and insulin during a standard OGTT in relation to current BPA and phthalate exposure. We conducted a thorough body compositional and metabolic examination of 107 healthy children with concomitant determination of urinary excretion of BPA and phthalate levels in full morning urine.

Our present findings that the group of children with the highest BPA levels had better insulin sensitivity and lower triglyceride levels is contradictive to most previous studies in children (6, 23) and do not support that concurrent BPA exposure has adverse effects on glucose homeostasis. However, due to the cross-sectional design of the present study, the temporal cause–and-effect relationship could not be determined. The fact that higher BPA exposure should have a positive influence on glucose metabolism seems unlikely. Thus, reverse causality – that the children with the healthier and more active lifestyle were more exposed to BPA. This could hypothetically be through consumption of more food and drinks contained in BPA-coated packaging to compensate for the higher energy expenditure of the more active lifestyle.

Previous cross-sectional studies showing higher prevalence of obesity and diabetes in adults (4, 5, 24, 25) and obesity in children (23) with higher BPA exposure may also be due to reverse causality. Exposure to BPA is mainly through intake of food and drinks contained in BPA-coated packaging – and exposure to BPA will therefore on average be higher with higher caloric intake. Thus, high BPA excretion may simply be a biomarker for intake of excess calories in these studies. To avoid this confounding, detailed and reliable data on the dietary sources of BPA exposure, calorie intake and energy expenditure such as physical activity is of paramount importance and have not been taken into account in previous studies.

While human studies are conflicting regarding the potential harmful effect of BPA on metabolism, experimental rodent studies show a more consistent pattern of BPA having adverse effects on the metabolic system, though the exact effect of BPA is yet to be determined. In mice that received a high-fat diet, BPA exposure was associated with more severe insulin resistance (26) and increased body weight and fat mass (27). Another study found that BPA did not induce body weight or fat mass but was instead disrupting glucose homeostasis, and this in fact was aggravated by a high-fat diet (28). Altogether, this could suggest that high doses of BPA may have an independent role in promoting obesity and/or insulin resistance in mice – though it is important to note, that in these experimental studies, the rodents are treated with about a thousand times greater dose of BPA compared to the exposure of the children in our clinical study.

It has been suggested that the harmful effect of BPA may be limited to a window of susceptibility, proposing that prenatal exposure in children (29) or exposure during pregnancy (30) may have long-term deleterious consequences in mice. However, no human data are available to confirm these suggestions. We evaluated current exposure levels with current body composition and glucose metabolism in children and adolescents and found no evidence that current exposure of these metabolites has an harmful effect on body composition or glucose metabolism at the time of exposure in healthy children, though further investigation is needed to prove of any long-term effects.

The lack of solid human data to confirm the results from the experimental models still renders the question if BPA has adverse effects on body composition and glucose metabolism in humans unanswered. Prospective studies with repeated measurement of BPA and metabolic risk profiling are needed before firm conclusion can be made.

An association between phthalate exposure and obesity has been suggested, but controversy exists. One study based on NHANES data showed a positive association between urinary levels of some phthalates and waist circumference in men but not in women (10). A study on the Swedish population showed the same, though the association was only seen in women but not in men (31). Similarly, no consistent pattern is evident when assessing associations between phthalates and diabetes or markers of glucose metabolism (8, 32, 33). These inconsistencies could be due the same abovementioned reasons – the existence of reverse causality and the lack of dietary source of phthalate exposure. In the present study, no associations were found between phthalate exposure and either adiposity or glucose metabolism.

The strength of this investigation is the unselected sample of 107 well-characterized children. The primary limitation of this study is its small size, and the cross-sectional design, which cannot show the temporal pattern of exposure and disease. Furthermore, our study population consists of healthy and normal-weight children, and we may therefore not have been able to observe the associations presented in other studies including overweight and obese subjects. In addition, since these chemicals do not persist *in vivo*, a first morning urine sample will only be suggestive of exposure for the previous day and may not give an accurate measure of long-term exposure. A high intra-individual day-to-day variation in urinary BPA excretion exists (34), which may lead to risk of exposure misclassification.

## Conclusion

In conclusion, our study on healthy, normal-weight children do not support that current exposure to phthalates and bisphenols is associated with adverse effects on insulin resistance or adiposity. Other cross-sectional studies showing positive association between BPA and obesity might be confounded by lack of data on caloric intake and energy expenditure. However, studies on murine models continue to show adverse health effect. Therefore, further prospective studies with serial measurements of the chemicals of interest, detailed measures of nutrient intake and energy expenditure, as well as long-term follow-up are greatly warranted.

## Declaration of interest

The authors declare that there is no conflict of interest that could be perceived as prejudicing the impartiality of the research reported.

## Funding

Research Fund of Capital Region of Copenhagen; Research Fund of Rigshospitalet; Kirsten og Freddy Johansen Foundation.

## Consent

Consent has been obtained from each patient or subject after full explanation of the purpose and nature of all procedures used.
